# Supplementary material for: Somatostatin Receptor 2 Expression Profiles and Their Correlation with the Efficacy of Somatostatin Analogues in Gastrointestinal Neuroendocrine Tumors
Source: Cancers (Basel). 2022 Feb 2;14(3):775. doi: 10.3390/cancers14030775 (PMC8834049; doi:10.3390/cancers14030775)
Supplement: Supplementary file 1 [file cancers-14-00775-s001.zip › Figure S1.pdf]

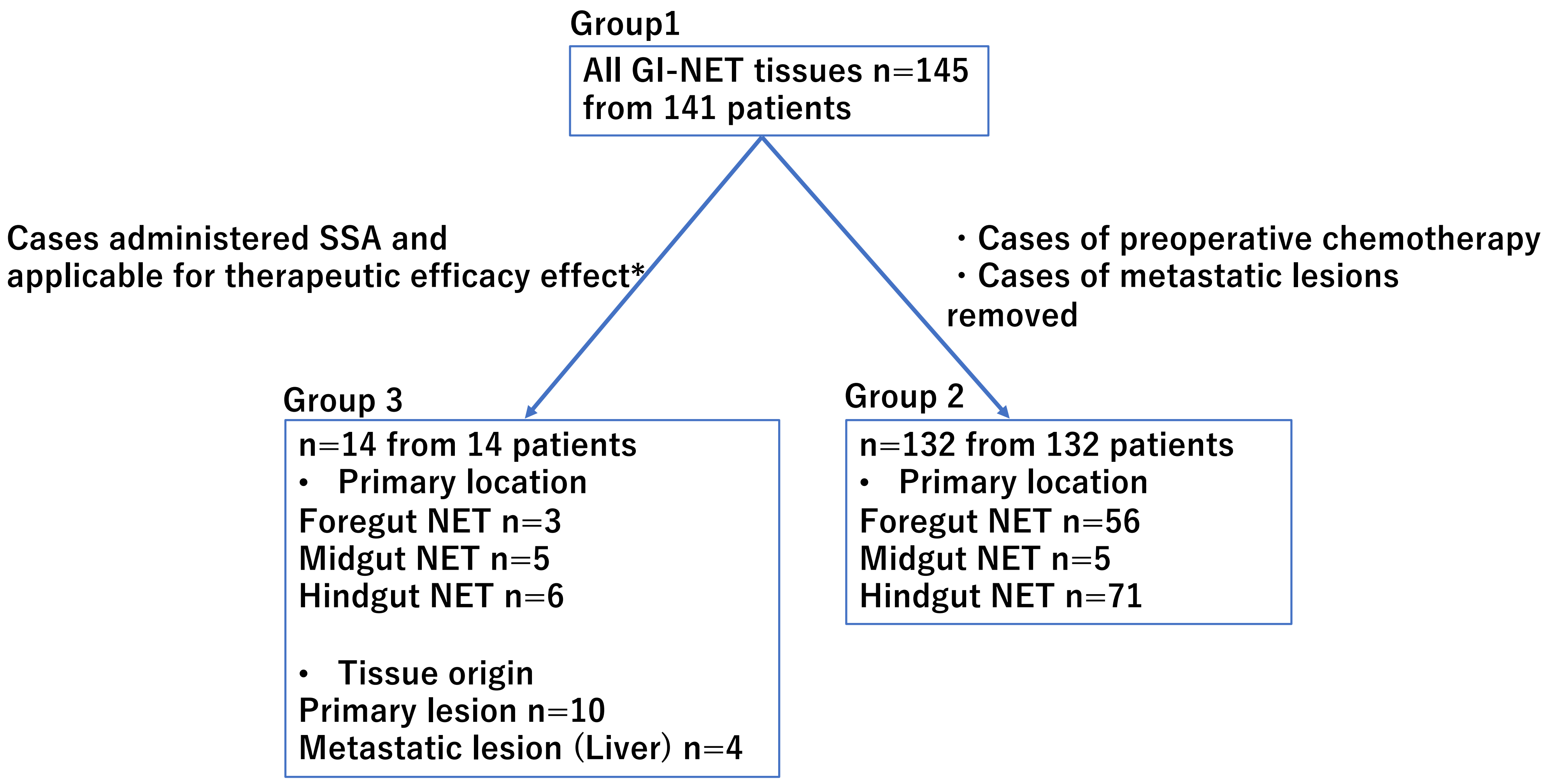


* When multiple tissue samples were available from a single patient, the tumor tissue sample that was therapy-naïve, collected shortly after SSA administration, or from the primary site was selected.
