# Supplementary figures and images for: Somatostatin Receptor 2 Expression Profiles and Their Correlation with the Efficacy of Somatostatin Analogues in Gastrointestinal Neuroendocrine Tumors
Source: Cancers (Basel). 2022 Feb 2;14(3):775. doi: 10.3390/cancers14030775 (PMC8834049; doi:10.3390/cancers14030775)

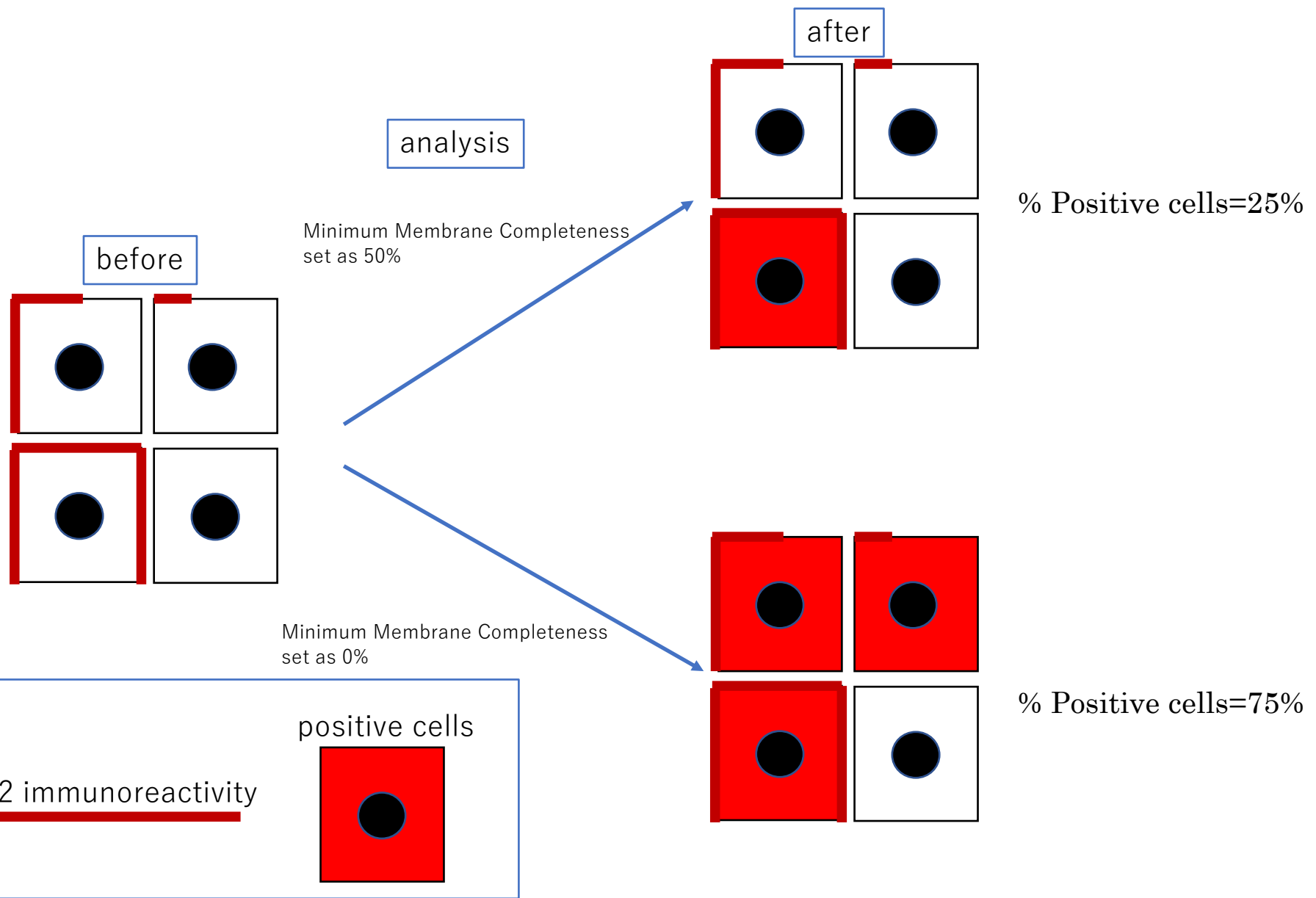

Supplement: Supplementary file 1 [file cancers-14-00775-s001.zip › Figure S2.pdf]

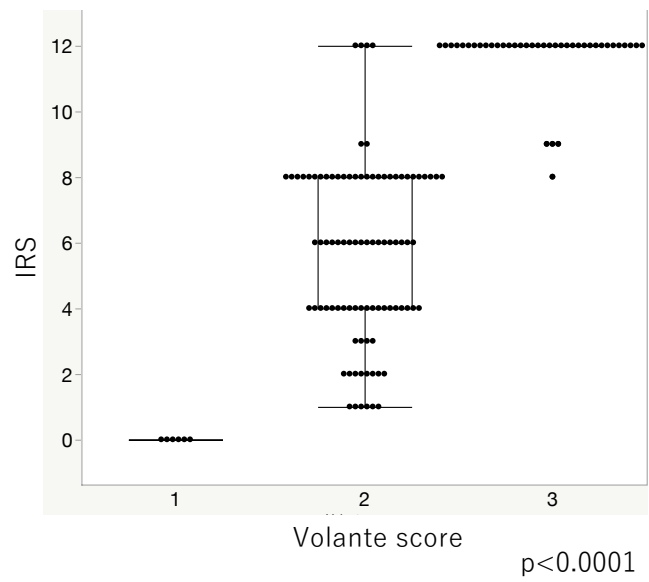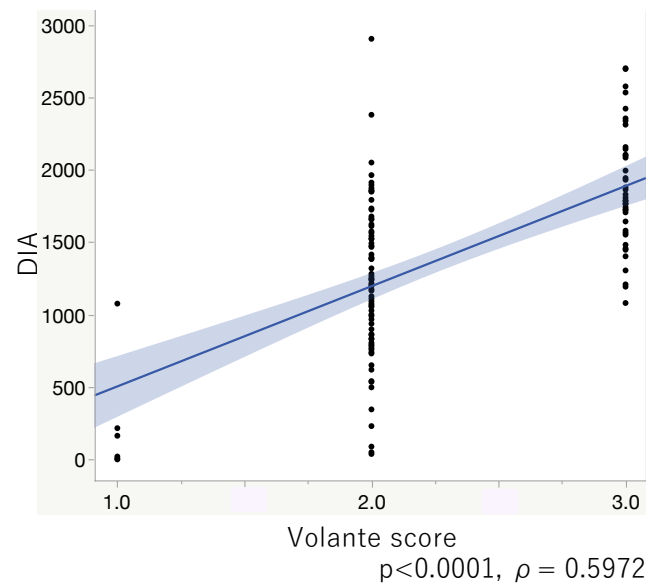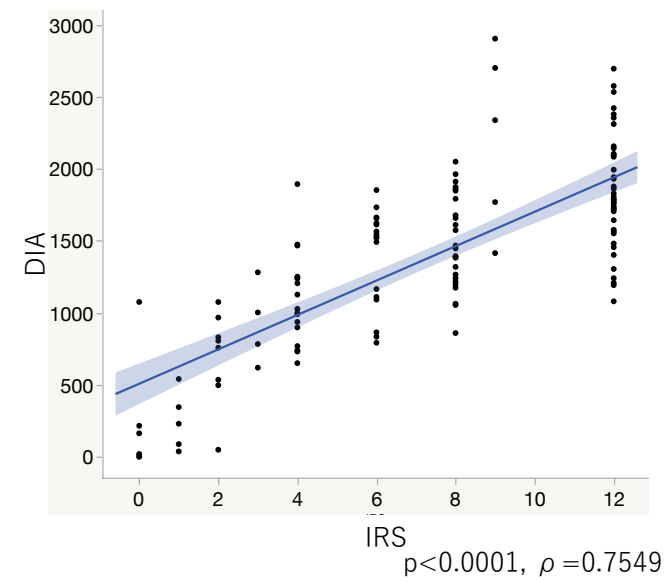

Supplement: Supplementary file 1 [file cancers-14-00775-s001.zip › Figure S3.pdf]

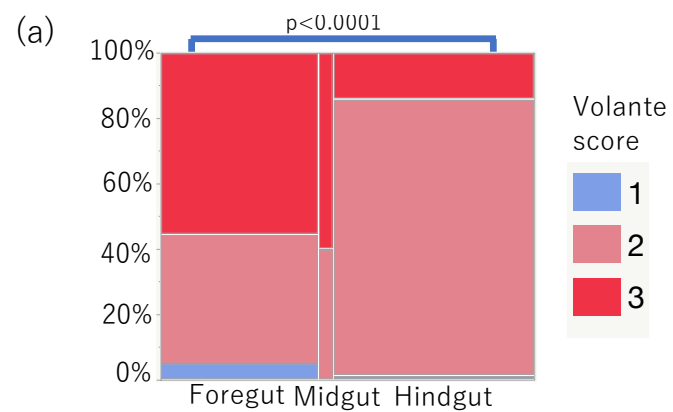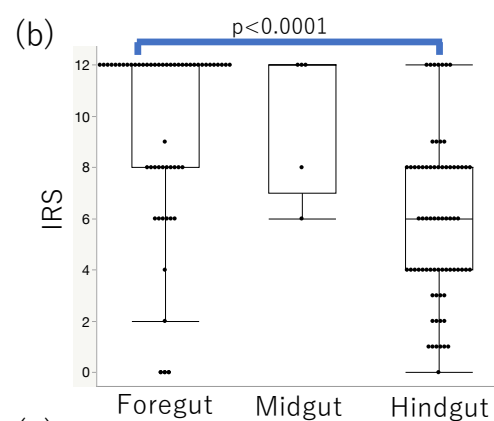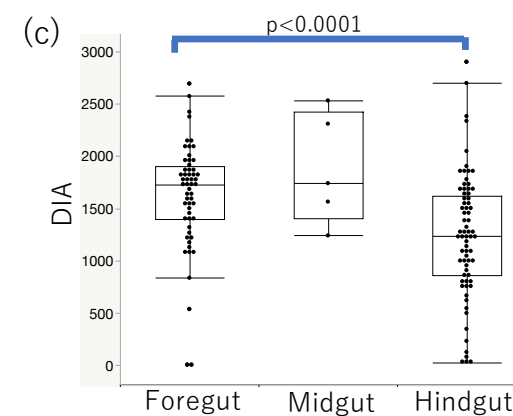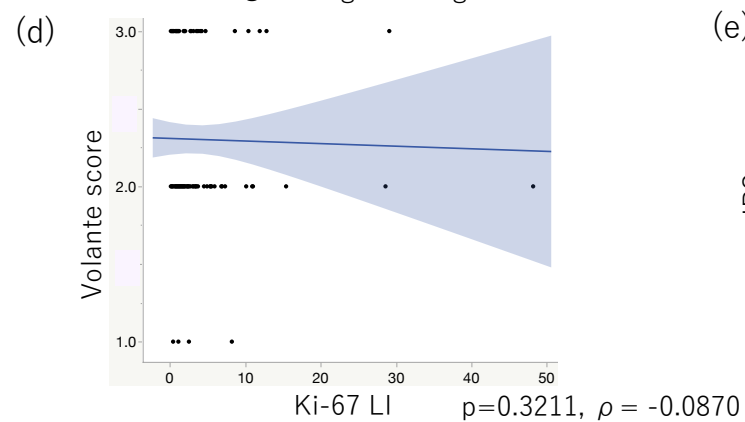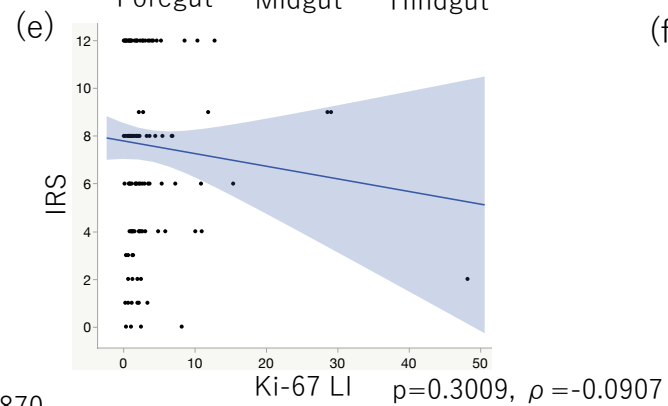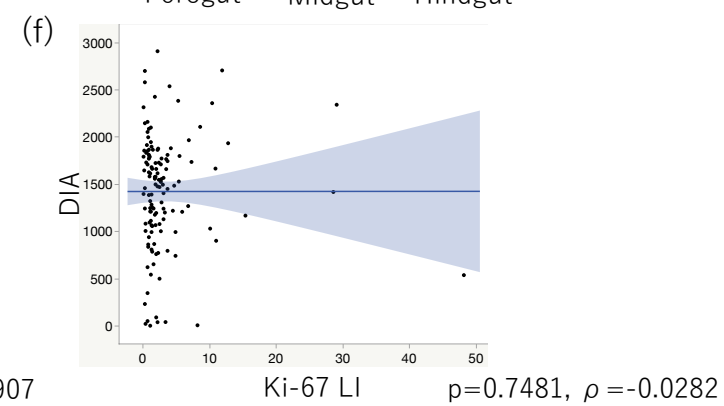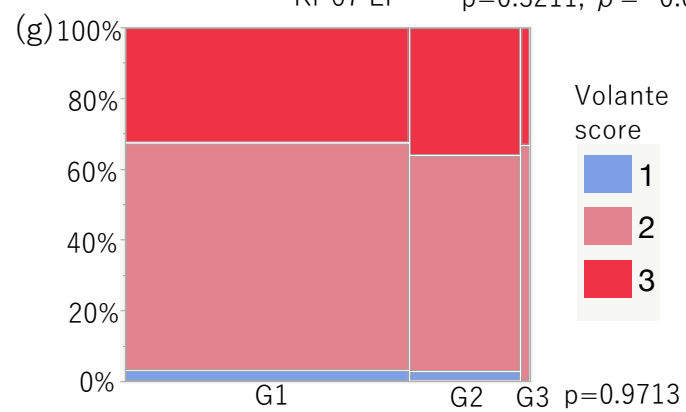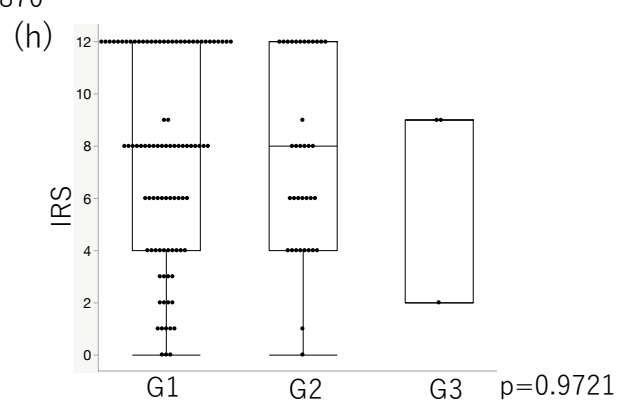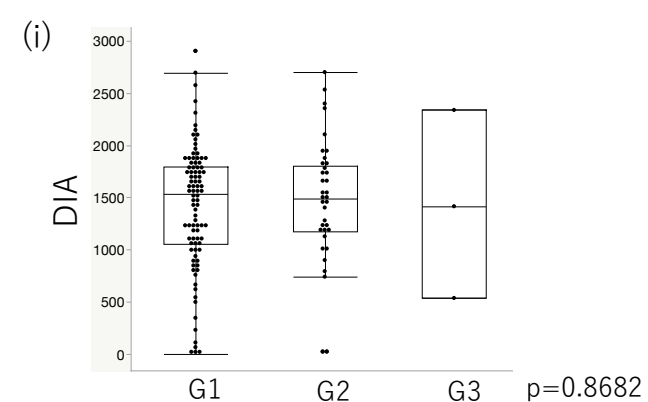

Supplement: Supplementary file 1 [file cancers-14-00775-s001.zip › Figure S4.pdf]

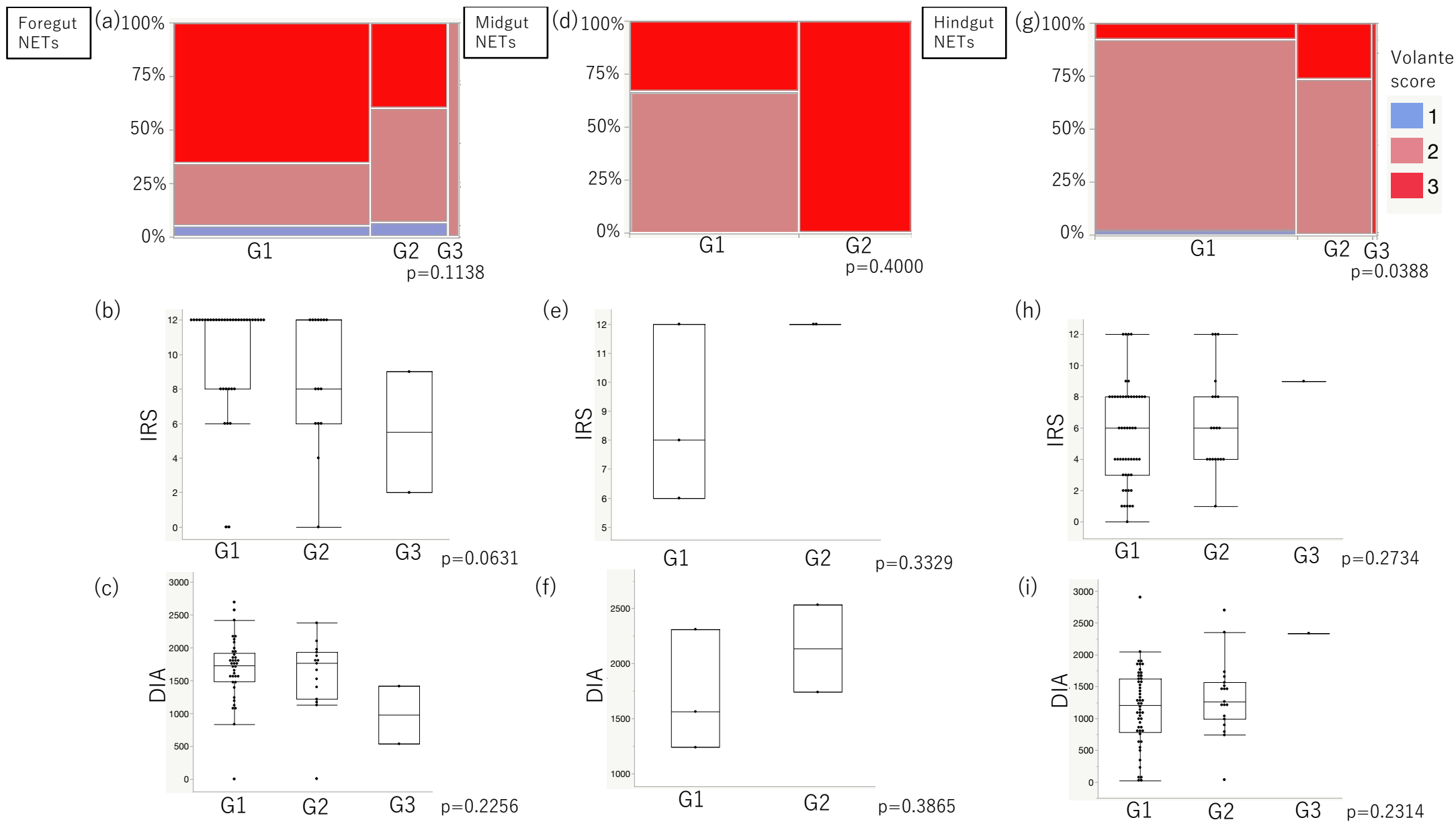

Supplement: Supplementary file 1 [file cancers-14-00775-s001.zip › Figure S5.pdf]
